# Case Report: retained gutta-percha as a cause for persistent maxillary sinusitis and pain

**DOI:** 10.12688/f1000research.3791.2

**Published:** 2014-04-29

**Authors:** Benjamin L. Hodnett, Berrylin Ferguson

**Affiliations:** 1 Department of Otolaryngology, University of Pittsburgh Medical Center Eye and Ear Institute, Pittsburgh, PA, 15213, USA; 2 Department of Otolaryngology, University of Pittsburgh Medical Center, Pittsburgh, PA, 15219, USA

## Abstract

Dental sources of infection can produce acute and chronic maxillary sinusitis. In some cases, the source of the infection may be related to the presence of endodontic materials in the oral cavity. In this article, we report a case of retained gutta-percha in the maxillary sinus resulting in chronic sinusitis.

## Introduction

Foreign bodies in soft tissues can often occur as a result of trauma. There are multiple case reports in the otolaryngology literature of projectiles that have become lodged in the paranasal sinuses ^1^. Retained packing gauze from endoscopic sinus surgery has also been reported in the paranasal sinuses ^2^. In the endodontic literature, many reports and reviews have described various materials that can cause disease of the maxillary sinuses ^3^. In this case report, we present an interesting case of a patient who presented with recalcitrant maxillary sinusitis that was ultimately found to be related to retained endodontic material in her maxillary sinus.

## Case report

A 26-year-old Caucasian woman presented with a chief complaint of left-sided maxillary pain with intermittent, discoloured nasal drainage. Seven years prior to current presentation, the patient had reported a history of headaches, nasal congestion and bilateral discolored drainage refractory to prednisone, antibiotics, and endoscopic sinus surgery at an outside facility. Revision endoscopic sinus surgery one year after the initial surgery provided partial relief. Two years prior to current presentation, her left maxillary pain recurred, and a computed tomography (CT) scan revealed a left wisdom tooth projecting into her maxillary sinus. Following wisdom teeth extraction, she had marked improvement in pain and nasal drainage; however 8 weeks later, left maxillary pain returned and was associated with left-sided yellow nasal discharge. Her oral surgeon discovered an infection in the molar adjacent to the extracted wisdom tooth and performed a root canal.

**Figure 1.**
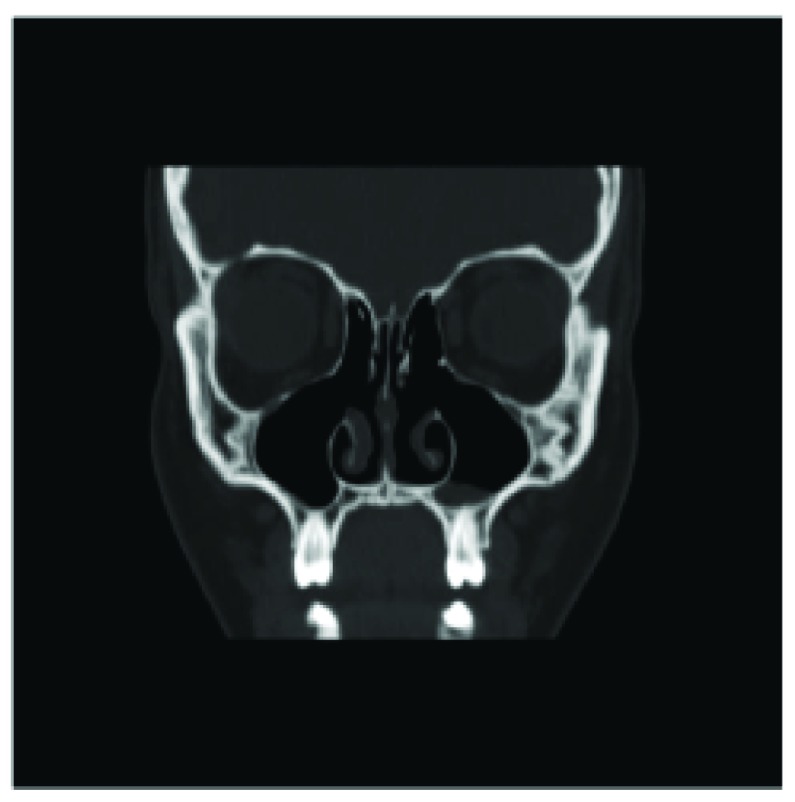
Non-contrasted coronal CT scan from two years prior to patient presentation. Only mild mucosal thickening of the floor of the left maxillary sinus and post-surgical changes related to endoscopic sinus surgery were noted.

Two weeks following the root canal, she presented with continued left maxillary tooth pain and left-sided discolored nasal discharge. Extraction of the molar failed to resolve her pain, and she was subsequently referred to the facial pain/headache clinic by her dentist where she was prescribed gabapentin 300 mg TID, which was ineffective. Her dentist also prescribed her multiple courses of clindamycin 150 mg TID and guaifenesin 600 gm QID, which would improve her pain and discoloured drainage. However, her symptoms would return after completing the antibiotics. On examination with rigid endoscopy, she had widely patent maxillary, ethmoid, and frontal sinus ostia with no purulence or polyposis. On flexible endoscopic examination of the floor of her left maxillary sinus, white to slightly yellow mucus was found. Aspiration of the mucus relieved the patient’s discomfort. Maxillary sinus cultures revealed few polymorphonuclear leukocytes, few mononuclear cells, and no microorganisms. Her most recent CT scan from two years prior to presentation at our facility was remarkable for minimal mucosal thickening of the floor of the left maxillary sinus was otherwise normal ( Figure 1). An occult dental infection was considered high in the differential diagnosis; however, because no actual dental infection could be demonstrated, a medial maxillectomy was considered in order to facilitate topical washing of the left maxillary sinus. Follow-up appointments in the facial pain/headache clinic found “some features of migraine, but it is unclear whether headaches represent primary or secondary headaches with migraine features”. Repeat evaluations by her dentist found no evidence of a dental infection. Because of past improvement on an 8-week course of clindamycin 300 mg TID, she was prescribed a 12-week course of clindamycin 300 mg TDI before surgery was considered, and she was referred to an infectious disease specialist.

**Figure 2.**
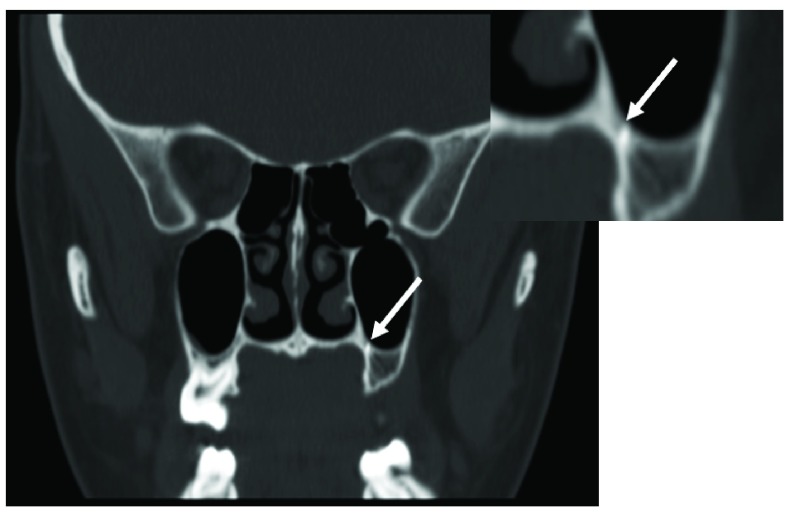
Repeat non-contrasted coronal CT scan. Retained gutta-percha (white arrow) noted in left maxillary sinus. Inset in the top right corner reveals a zoomed image of the gutta-percha.

Clindamycin initially improved her symptoms, but midway through the course her congestion returned. A bone scan was positive in the region of the left maxilla; however, a repeat indium scan was negative. Her CT scan was repeated and she was referred to an oral surgeon for evaluation ( Figure 2– Figure 4).

**Figure 3.**
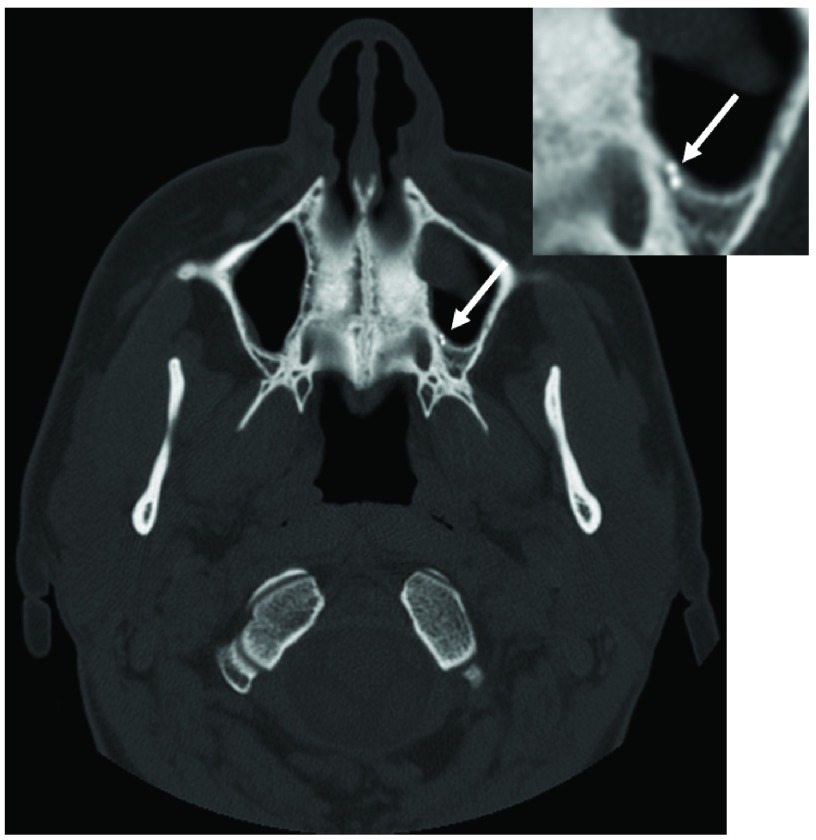
Axial section of non-contrasted CT scan shows two retained remnants of gutta-percha (white arrow) in the left posterior maxillary sinus. Inset in the top-right corner reveals a zoomed image of the two gutta-percha remnants.

**Figure 4.**
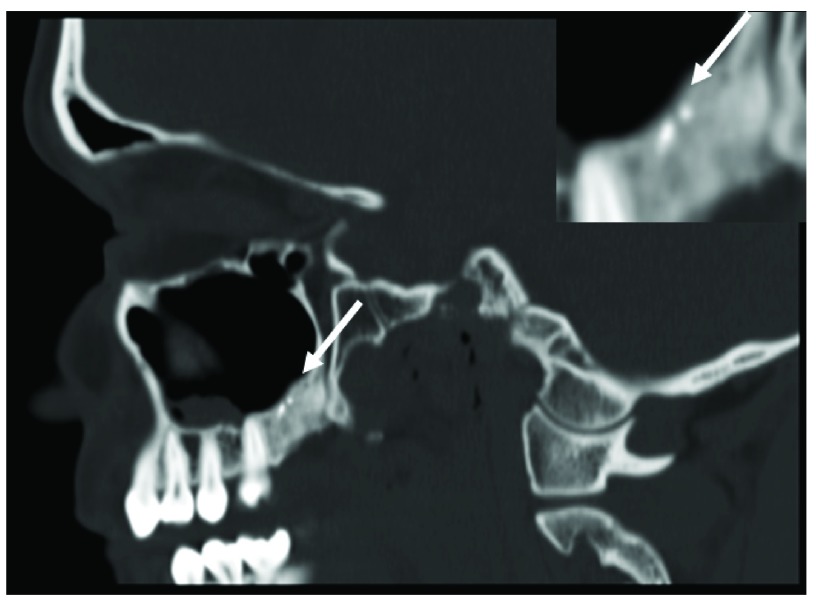
Sagittal section of non-contrasted CT scan shows two retained remnants of gutta-percha (white arrow) in the left maxilla in the location of previous dental extractions. Inset in the top-right corner reveals a zoomed image of the two gutta-percha remnants.

The CT scan was evaluated by an oral and maxillofacial surgeon who noted that there were two small remnants of the prior left maxillary root canal that had been performed two and half years ago ( Figure 2– Figure 4). A combined endoscopic and Caldwell-Luc approach under computer-assisted navigation to drill out retained gutta-percha in the maxillary sinus resolved the patient’s pain and drainage immediately, without recurrence at her three month follow-up. No pathological specimens were available for analysis as the gutta-percha remnants were drilled out during the surgery.

## Discussion

Gutta-percha, a product of tropical rubber plants, has been used since the mid-1800s as an endodontic filling material following root canal procedures ^4^. The chemical structure of gutta-percha is a trans-isomer of poly-isoprene, or natural rubber; however it is more crystalline than natural rubber and often is formulated with medications such as zinc oxide, iodoform, chlorhexidine and calcium hydroxide, which contribute to both the antibacterial and antifungal activity ^4– 7^. Animal studies have shown that gutta-percha becomes encapsulated by fibrous connective tissue with little inflammatory reaction ^8^.

Maxillary sinus complications from gutta-percha from root canals are rare. According to a previous case report, gutta-percha from a maxillary tooth root canal can migrate to and obstruct the maxillary ostium ^9^. Gutta-percha has also been reported to migrate into the ethmoid sinus ^10^. In the current patient, retained gutta-percha in the maxillary sinus resulted in chronic inflammation and a persistent sinusitis-type picture with nasal congestion, pain and drainage. Her symptoms preceding the sinus surgery may or may not have been related to dental infection, but she clearly improved following wisdom teeth extraction, and she relapsed due to the infection of the adjacent molar. The retained gutta-percha prevented the resolution of infection and symptoms, even following extraction of the affected tooth. Partial improvement with antibiotics directed to usual dental pathogens provided some evidence that the etiology of her symptoms was a dental infection. In patients with persistent sinusitis and a history of endodontic procedures, an evaluation for dental materials retained in or near the sinuses may be warranted to rule out an additional source of infection. Removal of these retained dental materials may require an external approach with drilling, which can be facilitated by endoscopic visualization through the Caldwell-Luc procedure.

A review of the otolaryngology literature did not provide any additional case reports on retained gutta-percha. In fact, only two case reports were found in the oral and maxillofacial surgery literature that described retained gutta-percha in the paranasal sinuses. In the oral surgery and endodontic literature, there are multiple reports of aspergillosis occurring in the maxillary sinus as a result of overextension of root canals of maxillary teeth, especially using materials containing zinc oxide or formaldehyde ^3, 11^. One case series of aspergillosis of the maxillary sinus was found in the otolaryngology literature. In this series, 85 cases of aspergillosis of the maxillary sinus in non-immunosuppressed patients were reviewed. Of these, 94% presented evidence of a radio-opaque foreign body in the maxillary sinus, with 85% of the cases related to endodontic dental paste ^12^.

Our case report highlights the importance of investigating alternative sources of infection in cases of recalcitrant sinusitis. Dental sources of infection as well as retained or overextended endodontic materials should be investigated in patients with unexplained, chronic sinusitis.

## Consent

Written informed consent for publication of clinical details and clinical images was obtained from the patient.
