# Supplementary material for: Case Report: retained gutta-percha as a cause for persistent maxillary sinusitis and pain
Source: F1000Res. 2014 Apr 29;3:81. Originally published 2014 Mar 31. [Version 2] doi: 10.12688/f1000research.3791.2 (PMC3999928; doi:10.12688/f1000research.3791.2)
Supplement: Peer review history

# Peer review: Referee response for version 2

**DOI:** 10.5256/f1000research.4326.r4889

**Published:** 2014-05-27

**Authors:** Michael P. Platt

**Affiliations:** 1 Department of Otolaryngology, Boston University School of Medicine, Boston, MA, USA

I have read this submission. I believe that I have an appropriate level of expertise to confirm that it is of an acceptable scientific standard.

# Peer review: Referee response for version 2

**DOI:** 10.5256/f1000research.4326.r4584

**Published:** 2014-04-29

**Authors:** Alkis Psaltis

**Affiliations:** 1 Department of Otolaryngology, Royal Adelaide Hospital, Adelaide, SA, Australia

Thank you for the additional images which are easier to follow and for the clarification on why there was an absence of radiological inflammation. This indeed is an interesting case report.

I have read this submission. I believe that I have an appropriate level of expertise to confirm that it is of an acceptable scientific standard.

# Peer review: Referee response for version 1

**DOI:** 10.5256/f1000research.4060.r4315

**Published:** 2014-04-16

**Authors:** Michael P. Platt

**Affiliations:** 1 Department of Otolaryngology, Boston University School of Medicine, Boston, MA, USA

This is an interesting case that highlights the importance of identifying odontogenic causes for chronic maxillary sinusitis.

I have read this submission. I believe that I have an appropriate level of expertise to confirm that it is of an acceptable scientific standard.

# Peer review: Referee response for version 1

**DOI:** 10.5256/f1000research.4060.r4314

**Published:** 2014-04-09

**Authors:** Alkis Psaltis

**Affiliations:** 1 Department of Otolaryngology, Royal Adelaide Hospital, Adelaide, SA, Australia

The value of single patient case reports to the scientific literature is debatable. Other than facilitating the documentation of rare conditions or experimental techniques, they are usually just articles of interest. Having said this, this well written article describing an odontogenic cause of refractory left maxillary sinusitis does highlight the importance of a broad differential when treating patients with sinus symptoms. My only criticism is the imaging provided and its purpose. The arrows used to highlight the foreign material, particularly in the coronal CT scan, appear to be pointing into space. Furthermore, there is little evidence of mucosal inflammation or osteitis, that one would expect to see in a patient with a foreign body reaction with symptoms of nasal discharge. Nevertheless, the significant improvement the patient experienced on removal of the retained gutta-percha does suggest that this was indeed the underlying cause of her symptoms.

I have read this submission. I believe that I have an appropriate level of expertise to confirm that it is of an acceptable scientific standard, however I have significant reservations, as outlined above.

# Response

**Published:** 2014-04-11

**Authors:** Benjamin Hodnett

**Affiliations:** UPMC, USA

This CT scan was performed at the completion of an antibiotics course, which may explain the lack of inflammation. The radiology reports minimal inflammation as well.  The patient had been on multiple courses of antibiotics with symptomatic improvement for a period of time after each treatment. The lack of osteitis is interesting. However, as stated in the discussion, gutta-percha in animals becomes encapsulated by fibrous connective tissue with little inflammatory response. The mechanism by which an encapsulated foreign body with little surrounding inflammation may precipitate a chronic sinus infection is indeed unusual.  We will also provide additional images which should better illustrate the foreign material.

# Peer review: Referee response for version 1

**DOI:** 10.5256/f1000research.4060.r4316

**Published:** 2014-04-09

**Authors:** Rakesh K Chandra

**Affiliations:** 1 Department of Otolaryngology, Northwestern University, Chicago, Il, USA

An interesting case that creates awareness about odontogenic sources of maxillary sinusitis. the authors should describe any histopathology findings - what type of inflammation was seen - eg  neutrophilic, eosinophilic, histiocytes, foreign body giant cell, etc.

I have read this submission. I believe that I have an appropriate level of expertise to confirm that it is of an acceptable scientific standard.

# Response

**Published:** 2014-04-11

**Authors:** Benjamin Hodnett

**Affiliations:** UPMC, USA

No pathological specimen was available. The foreign material was embedded in the bone and drilled out by the oral surgeons.
